# History and Experience: A Survey of Traditional Chinese Medicine Treatment for Alzheimer's Disease

**DOI:** 10.1155/2014/642128

**Published:** 2014-01-29

**Authors:** Ping Liu, Mingwang Kong, Shihe Yuan, Junfeng Liu, Ping Wang

**Affiliations:** ^1^Key Laboratory of Traditional Chinese Medicine Resource and Compound Prescription of the Ministry of Education, Hubei University of Chinese Medicine, Wuhan 430065, China; ^2^Key Laboratory of Senile Dementia (Xing Nao Yi Zhi), State Administration of Traditional Chinese Medicine of the People's Republic of China, Hubei University of Chinese Medicine, Wuhan 430065, China

## Abstract

Traditional Chinese medicine (TCM) is practiced in the Chinese health care system for more than 2,000 years. In recent years, herbal medicines, which are used to treat Alzheimer's disease (AD) in China based on TCM or modern pharmacological theories have attracted considerable attention. In this paper, we discuss etiology and pathogenesis of AD, TCM therapy, and herbal extracts for the treatment of AD. There is evidence to suggest that TCM therapy may offer certain complementary cognitive benefits for the treatment of AD. Chinese herb may have advantages with multiple target regulation compared with the single-target antagonist in view of TCM.

## 1. Introduction

Alzheimer's disease (AD) is a disease of chronic and progressive intelligence damage which is considered as the most common type of dementia among older people [1]. Its main manifestations are functional disorders of language, memory, cognition, emotion, character, and behavior in the elderly. At present, there is more than 36 million people who are currently estimated to have AD and this number is expected to be 118 million by the year 2050 [2]. In China, it is estimated that there are already approximately 9 million people affected by AD and this number is likely to be 27 million by the year 2050 [3, 4]. AD is developing a major problem of society and medical science, the therapy has become the key point of improving the life quality of the aged. Previous studies showed that pathological characterization of AD includes extracellular deposition of senile plaques; formation of intracellular neurofibrillary tangles; lesions of cholinergic neurons together with synaptic alterations in cerebral cortex, hippocampus, and other brain regions [5]. And it is acknowledged that multiple factors involved in the progress of AD are apoptosis, oxidative stress, mitochondrial dysfunction, inflammatory responses, and disturbance of energy metabolism homeostasis. Current clinical drugs administered to slow down the progress of the deterioration in AD patients include cholinesterase inhibitors and agonists of N-methyl-D-aspartate receptors (NMDA) [6, 7], but none of these therapies has profound effects on halting the advancement of AD.

In China, traditional Chinese medicine (TCM) has a long history of the treatment of AD. With thousands of years of medical practice, TCM has accumulated rich theories and a great deal of valuable experience in the prevention and treatment of AD [8]. According to TCM, prescriptions composed of complex variety of many different herbs are used to treat AD clinically, such as Six Flavors Rehmannia Pills (LiuWei DiHangWan), Nourish the Heart Decoction (Yang Xin Tang), and Gastrodia and Uncaria Drink (Tian Ma Gou Teng Yin) [9].

Meanwhile, lots of Chinese herbs including Acorus, Polygala, Ginseng, Atractylodes, *licorice*, Astragalus, Pinellia, Curcuma, Salvia, Ziziphus, Rehmannia, Lycium *fruit*, Cistanche, Dioscorea, and *hoelen* are being used in AD as a new pathway for improvement of memory and cognitive function [10, 11]. Recent studies showed that TCM has been widely investigated for the treatment of AD in China [12]. So, it's significant to understand the TCM therapeutics and the natural medicines from traditional medicinal plants for AD. In this respect, we discuss etiology and pathogenesis of AD, TCM therapy, and herbal extracts for the treatment of AD to show the complementary cognitive benefits for the treatment of AD.

## 2. Etiology and Pathogenesis for AD on TCM 

In TCM, AD is categorized as *chidai*, *jianwang, *or *daizheng*. Many disorders of memory and cognitive deficit are correlated to AD throughout TCM ancient literature. In the book *Jingyue Quanshu* (Collected Works of Zhang Jingyue; 1624 A.D.), there is a chapter on dementia (*chidai*) which describes the collapse of original qi (yuanqi) and the phlegm obstruction of the meridians and orifices as the pathogenesis of dementia. A formula is *Qi Fu Yin* for dementia, comprised of *ginseng (Renseng), cooked rehmannia (Shudihuang), Chinese Angelica (Danggui), ziziphus (Dazao), baked licorice (Gancao),* and* Polygala (Yuanzhi)*, which is the earliest known description in the world of a herbal therapeutic strategy for dementia and still in present use for AD [13]. The book *Bianzheng Lu *(Collected Works of Chen Shiduo; 1690 A.D.), said that “treating phlegm is treating dementia” and suggested formula *Su Xin Tang*, comprised of* ginseng (Rensheng), hoelen (Fulin), Pinellia (Banxia), Bupleurum (Chaihu), Coptis (hulian), evodia (Wuzhuyu), gardenia (Zhizi), aconite (Fuzi), Chinese Angelica (Danggui), peony (Shaoyao),* and* ziziphus (Dazao)* to treat AD [14].

According to the theory of TCM, the brain is an outgrowth of and is nourished by the kidney. Brain defects and deterioration of the brain may be prevented, limited, or halted by the ingestion of kidney tonics. And, the energy from the kidney, that is called kidney essence, can produce marrow including cerebral marrow, spinal cord, and bone marrow. As *Huangdi's Classics on Medicine* said: “the brain is sea of marrow” and “kidney stores essence to generate marrow” [15]. The cerebral marrow can nourish the brain and maintain the physiological functions of the brain. If the kidney essence is insufficient, the production of cerebral marrow will be reduced, leading to various symptoms, such as dizziness, amnesia, and retard response.

Meanwhile, memory, cognition, and wisdom are believed to become disordered if brain is blocked by phlegm obstruction of the channels according to TCM theory. Since “all prolonged diseases can be attributed to phlegm,” phlegm-dampness and turbidity obstructing the orifices are pathogenesis of AD. Once obstructed by the static phlegm-turbidity, the sea of marrow becomes turbid, qi movement fails in ascending and descending, the spirit of brain loses nourishment, original spirit gets blocked, intelligence gets damaged, and memory gets rotten. As a result, dementia will arise.

Based on long period of clinical practice, TCM considered that “the elderly person tends to have copious phlegm and kidney deficiency”, “kidney deficiency tend to mind insufficiency”, “Dementia tend to have copious phlegm”, and “Dementia Treated by Resolving Phlegm” [16]. Moreover, in TCM, AD is considered to be a holistic and may involve multiple causes. It is believed that the disease is highly correlated to the abnormal functions of other organs including the kidney, liver, heart, and spleen, although the pathological site of AD is in the brain. For example, AD patients who initially have kidney deficiency may also develop stagnation of phlegm leading to AD. Therefore, it is considered that kidney-essence weak and phlegm stasis become the important pathogenesis for AD on TCM [17–19].

## 3. Chinese Medicine Compound for the Treatment of AD 

Now, many Chinese medicine compounds have been applied to the therapy of AD with different medical emphasis subjecting to varied symptoms of AD. Some classical prescriptions have been used for thousands of years and are still in present use. For example, recent results indicated that Danggui-Shaoyao-San (DSS), comprised of* Chinese Angelica (Danggui), Chinese herbaceous peony (Shaoyao), hoelen (Fulin), white atractylodes (Baizhu), Rhizoma Alismatis (Zexie), and Rhizoma Chuanxiong (Chuanxiong), *ameliorates age-related memory dysfunction, modulates metabolism of monoamine neurotransmitters, and protects ultrastructure of the cortex. These findings suggest that DSS may be a useful therapeutic agent for AD. The mechanism might be related to ameliorate dysfunction of the central cholinergic nervous system and scopolamine-induced decrease in Ach levels. Accordingly, *in vitro* study, results showed that DSS significantly reduced the A*β*_25–35_ induced neuronal death and the lipid peroxidation. The DSS extract JD-30 also could ameliorate A*β*_25–35_ induced impairment of spatial learning and memory in mice as well as inhibition of long-term potentiation (LTP) in the hippocampus [20–24].

Yokukansan, comprised of* Acorus tatarinowii (Shichangpu), Polygala tenuifolia (Yuanzhi), hoelen (Fulin), Ginseng (Renseng),* has attracted attention to treat AD in recent times. The results of a recent pilot study indicated that yokukansan can alleviate the behavioral symptoms of frontotemporal dementia. It is suggested that yokukansan might be effective in the treatment of behavioral and psychological symptoms of dementia. And it is demonstrated that yokukansan could ameliorate learning deficits and noncognitive defects including a decrease in the anxiety and an increase in locomotor activity observed in Tg2576 mice [25–27]. Tateno et al. demonstrated that yokukansan inhibited A*β*-induced cytotoxicity in a primary culture of cortical neurons in rats [28, 29]. It is indicated that yokukansan provoked an anticytotoxic mechanism to protect against cell death induced by glutamate. It has also been demonstrated that yokukansan increased acetylcholine levels and acetyltransferase activity in experimental animals [30]. As suggested by Tabuchi et al., yokukansan ameliorates cognitive and noncognitive symptoms in AD experimental rats without any relationship to A*β* deposition [31].

Since 2001 our group has been performing research on reinforcing kidney essence, removing phlegm, and promoting mental therapy in AD. In clinical study, sixty AD patients consistent with enrollment standards were divided into Bushenhuatanyizhi instant granules (BHY) group and the piracetam group using a randomized block design. BHY was composed of *Radix Polygoni Multiflori (Heshouwu), Rhizoma Panacis Japonici (Zhujieshen), Rhizoma Acori Tatarinowii (Shichangpu), Caulis Bambusae In Taeniam (Zhuru), Rhizoma Pinelliae (Banxia), Poria (Fuling), *and* Radix palygalae (Yuanzhi).* Patients in the BHY group took BHY instant granules (6 g, twice per day). The control group took piracetam (0.8 g, 3 times per day). There were twelve weeks in a course. Our results suggest that Bushenhuatanyizhi instant granules could significantly improve MMSE and ADL scores, increase SOD activity, and decrease the levels of LPO and TG after treatment. It showed that the therapy can improve cognitive function and daily life of AD patients. The mechanism of the therapy might be related to improving blood fat, scavenging free radicals and inhibiting lipid peroxides [32–38]. In addition, some other Chinese herbal formulae are also effective in treating AD. Therefore, it is demonstrated that using herbal medicine could be an alternative or complementary therapy for AD.

## 4. Chinese Materia Medica for the Treatment of AD 

In the book of *Sheng Nong Ben Cao Jing* (Han Dynasty, 1st-2nd centuries), as the earliest pharmacopeia existing on materia medica in China, some Chinese herbs such as *Polygala (Yuan Zhi), Ginseng (Ren Shen), Coptidis rhizoma (Huang Lian), and Longan (Long Yan) *were recorded to ameliorate the decline of people's memory. During recent decades, a growing number of preclinical and clinical studies have shown the efficiency of Chinese herb in AD treatment. A literature survey result showed that top 10 TCM herb ingredients including* Acorus gramineus Soland*, *Rhizoma Chuanxiong*, *Radix polygalae*, *Salvia miltiorrhiza Bge*, *Radix Rehmanniae Preparata*, *Radix Angelica sinensis*, *Lycium barbarum L.*, *Poria cocos*, *Radix astragali,* and *Fructus Corni* were prioritized for highest potential benefit to dementia intervention, related to the highest frequency of *Acorus gramineus Soland* in 1232 formulae collected from substantial documentary achievements [39, 40].

Of these, *Radix polygalae* is one of the most frequently used medicinal herbs for memory loss in TCM. *Radix polygalae* can significantly improve learning and memory and promote long-term potentiation (LTP) in the hippocampus* in vivo*. Its mechanism may be related to promoting neurogenesis and inhibiting acetylcholinesterase (AChE) activity [41, 42].

There is suggestive evidence that* Poria *could enhance hippocampal LTP and improve scopolamine-induced spatial memory impairment in rats. *Poria*'s cognitive action has been ascribed to AChE inhibition and bidirectional regulation of cytosolic free calcium [43, 44].

Recent studies showed that different fractions of* Rhizoma Acori Graminei* extract such as the soluble and essential oil fractions could improve the learning and memory abilities in AD mice induced by A*β*_1–42_. This effect might be related to the decrease in nitric oxide synthase (NOS) activity [45, 46].

In the mouse model of dementia, *Polygonum multiflorum* markedly improved learning memory, lowered the contents of lipofuscin and monoamine oxidase activity in the brain, and enhanced the activities of superoxide dismutase and catalase in the brain and NOS in the hippocampus [47, 48].

Today, huperzine A, an alkaloid from *Huperzia serrate* which is documented in Chinese medicine literature, is applied to improve memory and learning ability in AD patients [49]. In clinical study, huperzine A was administered to AD patients in 300 Ag/day doses for the first 2-3 weeks and then 400 Ag/day for the next 4–12 weeks. These patients significantly improved in their cognitive, noncognitive, and ADL functions [50, 51].

## 5. Conclusion

Alzheimer's disease (AD) is a complex, multifactorial, heterogeneous mental illness. In light of the pathogenic complexities of AD, it is probably unlikely that single-target drugs will achieve satisfactory curative effects. So, the treatment of AD remains a challenge in the modern medicine and current therapeutic modalities cannot successfully halt the progression of AD at an early stage.

In the long history of development of TCM, many single herbs and herbal compounds have been discovered and employed to treat AD in China. According to TCM theory, AD is assumed to be correlated with kidney essence vacuity and turbid phlegm blocking upper orifices. The whole cognitive function may worsen because of the aggravation of kidney essence vacuity, deficiency of blood and qi, and phlegm and may eventually lead to multiple cognitive domains impairment in the progress of AD [52].

Based on the history of application, there is strong clinical support that TCM could have a complementary and alternative role in preventing and treating AD. There is suggestive evidence that *Radix polygalae*, *Poria, Rhizoma Acori Graminei, and Polygonum multiflorum *are memory improving and were prioritized for highest potential benefit to AD treatment.

In recent years, scientists have isolated many active constituents from herbs, such as huperzine A, which can alleviate AD and neurodegenerative syndrome with fewer side effects than conventional drugs. Chinese herb may have advantages with multiple target regulation compared with the single-target antagonist in the view of TCM. And herbal drugs are relatively less toxic, can readily cross the BBB, and are bioavailable to exert multiple synergistic effects. Thus, single herb or compounds are regarded as promising drug candidates in treatment of AD. However, in the long run, efforts should be paid to understand what the active herbal components are in the formula, how they interact, and which herbs should be blamed for an adverse reaction if it happens.
